# Olfactory dysfunction is associated with mild cognitive impairment in community-dwelling older adults

**DOI:** 10.3389/fnagi.2022.930686

**Published:** 2022-08-08

**Authors:** Ai Che Yap, Rathi Mahendran, Ee Heok Kua, Weibiao Zhou, De Yun Wang

**Affiliations:** ^1^Department of Food Science and Technology, National University of Singapore, Singapore, Singapore; ^2^Department of Psychological Medicine, Mind Science Centre, Yong Loo Lin School of Medicine, National University of Singapore, Singapore, Singapore; ^3^Department of Psychological Medicine, National University Hospital, Singapore, Singapore; ^4^Department of Otolaryngology, Yong Loo Lin School of Medicine, National University of Singapore, Singapore, Singapore

**Keywords:** olfaction, smell identification, cognition, amnestic, mild cognitive impairment

## Abstract

**Background:**

Olfactory impairment is aging related and is associated with cognitive decline in older adults. However, it remains unclear if an olfactory impairment is associated with mild cognitive impairment (MCI) and the degree of impairment in the MCI subtypes.

**Materials and methods:**

In a cohort of community-living older adults aged 60 years and above, 670 participants were recruited from the Community Health and Intergenerational (CHI) Study. Olfactory function was assessed using a locally developed nine-item smell test in association with neurocognitive assessments. Analysis of covariance (ANCOVA) was used to examine the association of smell identification score and clinical cognitive status while considering demographic, clinical, and neuropsychological factors with Bonferroni correction for group comparisons.

**Results:**

Age-related smell detection between normal aging (NA) and MCI participants had no significant difference. The overall mean smell identification score in older adults was negatively correlated with age. The mean smell identification score of MCI participants was also not significantly different as compared with NA, but an amnestic MCI multiple domain (aMCI_MD) subgroup had significantly lower (impaired) mean smell identification scores compared with the NA and MCI single domain (amnestic and non-amnestic) group.

**Conclusion:**

This study demonstrated that olfactory identification impairment is a comorbidity in older adults with amnestic MCI (aMCI) and is a potential marker associated with an early stage of a neurocognitive disorder. The smell test could act as a screening tool to help in the early detection of smell impairment for heterogeneous syndromes of MCI among community-dwelling older adults. Clinicians and researchers could benefit from utilizing the locally developed smell test to screen their patients or research participants before the initiation of an appropriate health intervention or in a clinical trial.

## Introduction

The rapidly aging population worldwide has contributed to the annually accelerated dementia rates that are projected to triple from the current estimated over 50 million dementia cases by the year 2050 worldwide (World Health Organization, 2021). Alzheimer’s disease (AD) is one of the most common causes of dementia. Mild cognitive impairment (MCI) is a transition stage between normal cognition and AD. Mild cognitive impairment diagnosis may be reversible or convert to dementia over time (Jak et al., 2009). Approximately 15% of MCI cases were estimated to develop AD annually, and there is no effective treatment to cure AD (Murphy, 2019). In Singapore, the prevalence of cognitive impairment in local community-dwelling older Chinese adults was reported at 15.2% (Hilal et al., 2013). The current biomarkers to detect AD are invasive and expensive (e.g., cerebrospinal fluid measurement and neuroimaging) (Kotecha et al., 2018). A recent manuscript discussed efforts to increase awareness and reduce stigmatization of dementia and novel screening measures that could help to detect dementia earlier (Brai et al., 2021). Sensory impairment may be suggested as an alternative non-invasive biomarker to predict disease conversion. Commonly, more attention is paid to vision and hearing impairments as these senses significantly disrupt one’s daily life. However, our fifth sense, smell was always the least appreciated and underrated. This underrated sense has been proposed as a stronger predictive tool over the sense of hearing and vision for diagnosing AD-related dementia in the prodromal stage (Brai et al., 2020).

A meta-analysis study found that smell identification is impaired in those with MCI compared with healthy older adults (Roalf et al., 2017). Previous studies also reported that the MCI had impaired smell identification compared with the healthy control (Devanand et al., 2010; Liang et al., 2016; Tonacci et al., 2017). Longitudinal studies showed that cognitively intact older adults with smell identification impairment at baseline subsequently developed MCI in the follow-up (Wilson et al., 2007; Roberts et al., 2016; Palta et al., 2018). Therefore, the evidence suggests that smell identification tests have an effective role in the early detection and prediction of those cognitively intact older adults at risk of cognitive impairment in later life.

No local studies have assessed older adults’ smell function in general or the cognitively impaired population. To the best of our knowledge, the Community Health and Intergenerational (CHI) Study was the first comprehensive epidemiological study that included the objective measurement of olfactory health in assessing older adults in Singapore. Using the locally developed nine-item smell test, we aim to assess the smell identification ability of older adults and associate the smell test outcomes with their cognitive status. The differences in smell identification impairment between non-amnestic MCI (naMCI) and amnestic MCI (aMCI), between aMCI and normal aging (NA), and between naMCI and NA older adults were further analyzed, resulting in more explicit associations of olfactory and specific cognitive impairments.

## Materials and methods

### Study participants

**FIGURE 1:**
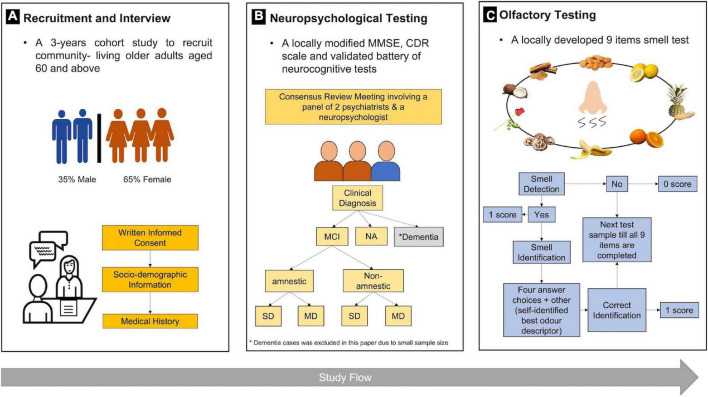
Study procedures for 670 older adults in **(A)** recruitment, **(B)** neuropsychological tests, and **(C)** olfactory testing. MMSE, Mini-Mental State Examination; CDR, clinical dementia rating; MCI, mild cognitive impairment; NA, normal aging; SD, single domain; MD, multiple domain.

The CHI Study is a large cohort study with a thousand bio-psycho-social profiles of community-living older adults aged 60 years and older. Written informed consent was taken, and the study site was located at the Hannah Senior Activity Centre (HSAC) in the central west district of Singapore. Participants were recruited using flyers, door-to-door visits, and word-of-mouth referrals. They took part in six visits for the whole study, with the first three covering interview questionnaires about demographic characteristics, personal health history, and health-related assessments and blood testing. The remaining visits were for psychological assessments, including neuropsychological tests of cognition and mood, dental examination, and cardiac assessments (Lee et al., 2020). The smell test was part of the assessment, and the data collection period was from March 2018 to August 2021. Participants diagnosed with dementia, Parkinson’s disease, stroke with current follow-up, history of head trauma, and chronic rhinosinusitis were excluded from this study. A total of 670 older adults who by Consensus Review were clinically diagnosed as either NA or MCI and had completed the olfactory test were included in this analysis (Figure 1). Approval to conduct this study was obtained from the Institutional Review Board of the National University of Singapore (NUS-IRB Reference code: H-17-047). The study procedures have been performed in accordance with the Declaration of Helsinki.

### Olfactory test

A locally developed, culturally adapted nine-item smell test was utilized to quantitatively assess the smell detection and identification performance of study participants. This smell test was validated in a separate study with 177 participants between 20 and 59 years (unpublished data). The locally familiar test items including almond, lemon, orange, pineapple, banana, coconut, rose, cinnamon, and mushroom were selected through a preliminary study for the most common odors used in smell identification test in Singapore. Synthetic sources and food-grade chemicals were purchased from Sigma-Aldrich (Steinheim, Germany). All the chemicals used were character impact compounds commonly described as almond odor [benzaldehyde (CAS number: 100-52-7; purity: ≥98%)]; lemon odor [citral (CAS number: 5392-40-5; purity: ≥96%)]; orange odor [(*R*)-(+)-limonene (CAS number: 5989-27-5; purity: ≥93%)]; pineapple odor [allyl hexanoate (CAS number: 123-68-2; purity: ≥98%)]; banana odor [isoamyl acetate (CAS number: 123-92-2; purity: ≥95%)]; coconut odor [gamma-nonanoic lactone (CAS number: 104-61-0; purity: ≥98%)]; rose odor [phenethyl alcohol (CAS number: 60-12-8; purity: ≥99%)]; cinnamon odor [*trans*-cinnamaldehyde (CAS number: 14371-10-9 purity: ≥98%)]; and mushroom [1-octen-3-ol (CAS number: 3391-86-4 purity: ≥98%)]. After a fixed sample pre-preparation time, each odor was delivered using a separated device with a constant and neat concentration at a time. The device was described in a filed invention disclosure to the National University of Singapore Industry Liaison Office (ILO) and is under the protection of intellectual property (ILO reference no. 2019-105). The duration of the smell test for each participant was 20–30 min.

The olfactory assessment was conducted in an interview format. Before test administration, participants were briefed by the examiner on instructions and demonstration of using the smell test setup. Participants held the device before releasing the channel containing a fixed amount of odor under their nostrils at a fixed distance. They were requested to sniff the presented odor continuously until they detected a scent. Each odor sample was given only once. They were required to respond to the detection task based on their perception of the presence of odor (yes or no). For smell detection, a successful detection was scored as one point on each test odor. It was followed by a smell identification task; the participants were required to identify the same test odor before choosing the narrated answer choices to select the target odor. They were allowed to provide the best descriptor if they were dissatisfied with the four answer choices provided. Similarly, successful identification of each odor was scored as one point. There was a time interval of at least 30 s between each odor presentation to the participant to prevent sensory fatigue. The smell detection score 0–9 was calculated with a total score of detected scent out of nine test items. The smell identification score was calculated as the total score of correct identification of each target odor.

### Cognitive function tests

Cognition was assessed with the locally modified mini-mental state examination (MMSE) scale (Feng et al., 2012), the clinical dementia rating (CDR) scale (Morris, 1993), and a validated battery of neurocognitive tests (Lee et al., 2012). The neurocognitive battery comprises five domains of verbal and learning memory (Rey Auditory Verbal Learning Test), attention and working memory (Digit Span Forward and Backward Task), divided attention and sequencing (Color Trails Test), visual-spatial abilities (Wechsler’s Block Design), and verbal fluency (Semantic Verbal Fluency-Animals). The diagnosis (NA, MCI, or dementia) was made at consensus review meetings involving two psychiatrists and a neuropsychologist based on Petersen’s MCI criteria (Petersen, 2004) and diagnostic and statistical manual of mental disorders (DSM-V) for dementia criteria (American Psychiatric Association, 2013). The clinical diagnosis of MCI was further categorized into amnestic and non-amnestic subtypes with single-domain and multi-domain deficits.

### Statistical analysis

The relevant data were presented as frequencies in percentage (%) or mean ± standard deviation (SD). All computations were performed using SPSS Version 22 (SPSS Inc., Chicago, IL, United States). The normality of data was examined using the histogram. Descriptive analyses were conducted using an independent *t*-test for continuous variables. Chi-square test or Fisher’s exact test, whichever was more appropriate, was used for categorical variables. Comparison of age group, gender, and cognitive function was analyzed by independent sample *t*-test or one-way ANOVA followed by *post-hoc* Tukey’s test for group comparison. For severely skewed data, a logarithmic transformation was applied before conducting further statistical analysis, and the geometric mean values were compared. Pearson’s correlation or Spearman’s correlation was performed to examine the association between olfactory, demographic, and neuropsychological performances. Analysis of covariance (ANCOVA) was used to compare the smell identification performance and cognitive status with adjustment of confounding variables, and *post-hoc* Bonferroni test was used for group comparison. A two-tailed *p*-value of less than 0.05 was considered statistically significant.

## Results

### Characteristics of participants

A total of 670 participants completed both olfactory and neurocognitive assessments. The demographic characteristics of the participants are summarized in Table 1. Among the 670 participants, 527 (78.7%) had cognitive scores within the normal range and were therefore diagnosed as NA, while 143 (21.3%) had MCI. Of the participants, 233 (34.8%) were men. The participants had a mean age of 67.9 ± 5.4 years, and the mean years of schooling were 13.3 ± 4.2 years. Six hundred and forty-five (96.3%) participants were ethnic Chinese residents. Their educational background, ethnicity, presence of chronic conditions, consumption of alcohol, and smoking history were not significantly correlated to the smell detection and identification score (Spearman’s correlation coefficient, *p* > 0.05). For the geriatrics depression scale (GDS) score and geriatrics anxiety inventory (GAI) score, there was no correlation for both scales with smell detection and identification score (Pearson’s correlation coefficient, *p* > 0.05). Within the MCI subtypes, 71 participants were diagnosed as naMCI with 57 single domain and 14 multiple domain deficits. Of the 72 aMCI participants, 47 were diagnosed with single domain deficits and 25 with multiple domain deficits.

**TABLE 1:** Demographic characteristics, self-reported medical history, lifestyle, and cognition of 670 participants.

Characteristics	NA (*n* = 527)	MCI (*n* = 143)	*P*-value or range
**Frequency number (%) or mean ± SD**			
Gender, male	176 (33.4)	57 (39.9)	0.166
Age (years)	67.9 ± 5.2	67.7 ± 5.9	0.620
Year of schooling	13.4 ± 4.3	12.9 ± 4.0	0.195
**Highest education level attained**			0.289
Nil	16 (3.0)	3 (2.1)	
Primary school	49 (9.3)	18 (12.6)	
Secondary school or ITE	116 (22.0)	29 (20.3)	
Pre-university or Diploma	146 (27.7)	49 (34.3)	
University	200 (38.0)	44 (30.8)	
Ethnicity, Chinese	511 (97.0)	134 (93.7)	0.295
Hypertension	166 (31.5)	53 (37.1)	0.228
Hyperlipidemia	230 (43.6)	71 (49.7)	0.218
Diabetes mellitus	53 (10.1)	18 (12.6)	0.363
Alcohol consumption	114 (21.6)	24 (16.8)	0.244
Smoking history	7 (1.3)	4 (2.8)	0.260
CDR	0.1 ± 0.2	0.2 ± 0.2	0.001
MMSE score	28.2 ± 1.8	27.5 ± 2.2	0.001
GDS	0.9 ± 1.6	1.4 ± 2.4	0.013
GAI	1.0 ± 2.3	1.3 ± 3.2	0.293
**MCI subtypes**			
naMCI_SD		57 (8.5)	60–86 (69.1 ± 6.7)
naMCI_MD		14 (2.1)	60–82 (70.4 ± 7.3)
aMCI_SD		47 (7.0)	60–79 (66.5 ± 4.3)
aMCI_MD		25 (3.7)	60–76 (64.9 ± 4.4)

NA, normal aging; ITE, Institute of Technical Education; CDR, clinical dementia rating; MMSE, mini-mental state examination (optimal suggested cutoff point = 26 for detecting early cognitive impairment) (Feng et al., 2012); GDS, geriatrics depression scale (cutoff score = 4 or 5 indicates depression symptoms) (Nyunt et al., 2009); GAI, geriatrics anxiety inventory (cutoff score = 10 or 11 indicates symptoms of anxiety) (Pachana et al., 2007); MCI, mild cognitive impairment; naMCI_SD, non-amnestic MCI single domain; naMCI_MD, non-amnestic MCI multiple domain; aMCI_SD, amnestic MCI single domain; aMCI_MD, amnestic MCI multiple domain; alcohol consumption indicated that participants drink at least once a month. Smoking history was based on the total number of current smokers.

### Smell detection scores

**FIGURE 2:**
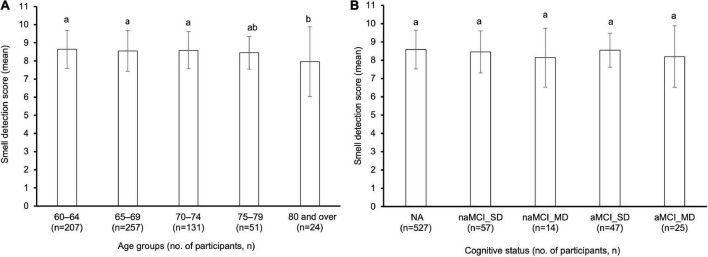
**(A)** Comparison of smell detection score between five age groups and **(B)** five clinical diagnosis categories in 670 older adults. Bars denote geometric mean and standard deviation. Different letters (i.e., a and b) above the bars indicated a significant difference between age groups or the clinical diagnosis groups (one-way ANOVA, *p* < 0.05). n, number of participants; NA, normal aging; MCI, mild cognitive impairment; naMCI_SD, non-amnestic MCI single domain; naMCI_MD, non-amnestic MCI multiple domain; aMCI_SD, amnestic MCI single domain; aMCI_MD, amnestic MCI multiple domain.

After logarithmic transformation, the age-related reduction in the geometric mean smell detection scores was statistically significant in the 80 years and over age group compared with the younger age groups from 60 to 75 years, as shown in Figure 2A (one-way ANOVA, *p* < 0.05). Meanwhile, no significant differences in the geometric mean smell detection scores were found between the NA and all MCI subtypes, as shown in Figure 2B (one-way ANOVA, *p* > 0.05).

### Smell identification score

**FIGURE 3:**
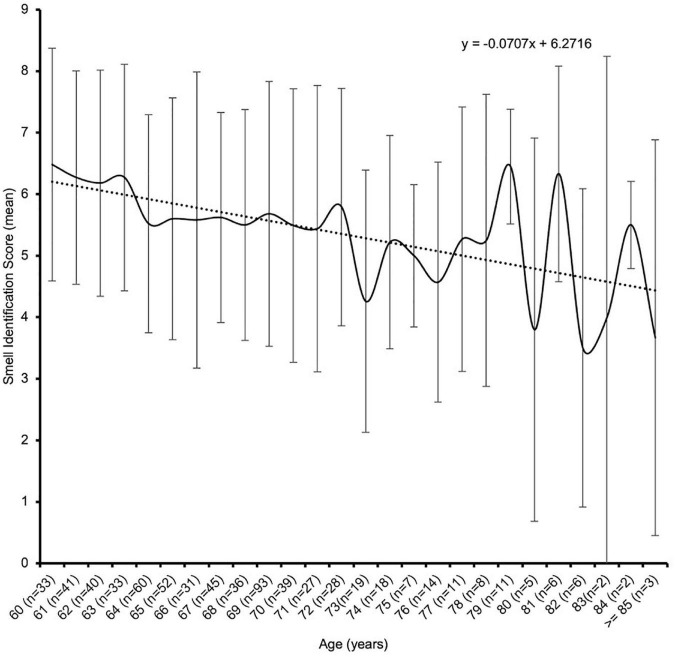
Age-related smell identification score of 670 older adults.

Older adults showed a slow and progressive decrease in the smell identification scores from 60 to 80 years and above. The smell identification score was weakly and inversely correlated with the ages of older adults using Pearson’s correlation coefficient analysis (*r* = –0.18, *p* < 0.01) as shown in Figure 3. As shown in Table 2, the smell identification score declined gradually with advancing age. The octogenarians had significantly lower smell identification scores (4.5 ± 2.6) than sexagenarians in the age group 60–64 years (6.1 ± 1.8) and 65–69 years (5.6 ± 2.0) (one-way ANOVA, *p* < 0.05). Generally, females (5.9 ± 1.9) significantly outperformed males (5.2 ± 2.1) in the smell identification test (independent samples *t*-test, *p* < 0.001). Among the females, significant poorer smell identification scores were achieved by those aged 80 and above years (4.2 ± 2.5) as compared with younger age groups from 75 years and below: 60–64 years (6.3 ± 1.8), 65–69 years (5.7 ± 2.0), and 70–74 years (6.0 ± 1.9) (one-way ANOVA, *p* < 0.05), while no significant difference was found in the smell identification scores of males across all age groups (one-way ANOVA, *p* > 0.05). The smell identification scores between the genders showed no significant difference after 75 years and above (75–79 years: 5.4 ± 1.9 vs. 5.1 ± 1.8 and 80 ≥ years: 4.2 ± 2.5 vs. 4.8 ± 2.9) (independent samples *t*-test, *p* > 0.05).

**TABLE 2:** Comparison of smell identification score between age groups and gender in 670 older adults.

Age groups (years)	All participants	Males	Females
60–64	6.1 ± 1.8^a^	5.6 ± 1.9^a,B^	6.3 ± 1.8^a,A^
65–69	5.6 ± 2.0^a^	5.5 ± 2.1^a,A^	5.7 ± 2.0^a,A^
70–74	5.3 ± 2.1^ab^	4.4 ± 2.1^a,B^	6.0 ± 1.9^a,A^
75–79	5.3 ± 1.9^ab^	5.1 ± 1.4^a,A^	4.5 ± 1.9^ab,A^
≥80	4.5 ± 2.6^b^	4.8 ± 2.9^a,A^	4.2 ± 2.5^b,A^
Total	5.6 ± 2.0	5.2 ± 2.1	5.9 ± 1.9*

Data are expressed as mean ± SD. Different lower case letters in superscript (i.e., a and b) within the same column indicate significant differences between age groups in all participants or within a gender (one-way ANOVA, p < 0.05). Different capital letters in superscript (i.e., A and B) within a row indicate significant differences between gender within the same age group (independent samples t-test, p < 0.05). For instance, the first and second letters separated with a comma (e.g., “a, A”) on males or females categories show the statistically significant result from one-way ANOVA within the same column and independent samples t-test between the two groups of males and females within the same row, respectively.

Asterisk (*) indicates a significant difference between males and females in total age groups (independent samples t-test, p < 0.001.

**FIGURE 4:**
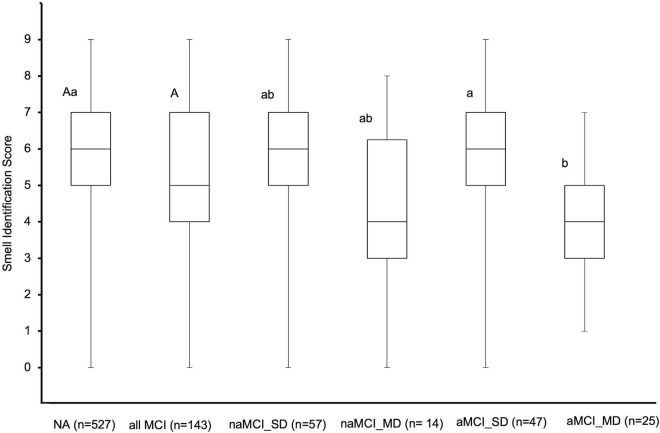
The measured mean smell identification score of the various cognitive status of 670 older adults. The capital letter “A” above the interquartile ranges denoted no significant difference between NA and total MCI participants (one-way ANOVA, *p* < 0.05). Different small letters (i.e., a, b) above the interquartile ranges denoted significant differences in mean smell identification scores between cognitive status. The first and second letters in the superscripts (e.g., “Aa” in NA category) show the two independent statistical significant results from a comparison of two groups’ differences (NA vs. all MCI) and five groups’ differences (NA vs. each MCI subtypes), respectively. NA, normal aging; MCI, mild cognitive impairment; naMCI_SD, non-amnestic MCI single domain; naMCI_MD, non-amnestic MCI multiple domain; aMCI_SD, amnestic MCI single domain; aMCI_MD, amnestic MCI multiple domain.

The comparison of the smell identification scores among the various clinical cognitive diagnosis state groups was shown in Figure 4. There was no significant difference in the smell identification scores between the NA and the total MCI participants (5.7 ± 2.0 vs. 5.3 ± 2.0, independent *t*-test, *p* > 0.05). Furthermore, no significant differences were detected in the mean smell identification scores between the NA (5.7 ± 2.0), amnestic MCI single domain (aMCI_SD) (5.8 ± 1.9), non-amnestic MCI single domain (naMCI_SD) (5.6 ± 2.0), and non-amnestic MCI multiple domain (naMCI_MD) (4.4 ± 2.3) participants (one-way ANOVA, *p* > 0.05). Interestingly, amnestic MCI multiple domain (aMCI_MD) (4.3 ± 1.6) had a significantly lower smell identification score than the NA (5.7 ± 2.0) and aMCI_SD (5.8 ± 1.9) groups (one-way ANOVA, *p* < 0.05). An ANCOVA was run to determine the association between smell identification score and cognitive status (NA and MCI subtypes). After adjustment for confounding variables (age, gender, year of schooling, MMSE score, hypertension, hyperlipidemia, diabetes, smoking history, alcohol consumption, GDS score, and GAI score), there was a statistical significance in smell identification score between cognitive status, *F*(4, 654) = 4.44, *p* < 0.01, *partial* η^2^ = 0.03, as shown in Table 3. Analysis of covariance with Bonferroni adjustment for multiple comparisons revealed that the aforementioned results remain statistically significant. In addition, a statistically significant difference between naMCI_SD group and aMCI_MD group was observed (mean difference = 1.45, 95% CI [−0.10, −2.79], *p* < 0.05) after covariates adjustment.

**TABLE 3:** Adjusted and unadjusted mean of smell identification score for different cognitive states.

	Unadjusted		Adjusted	
**Clinical diagnosis**	**Mean**	**SD**	**Mean**	**SE**
NA (*n* = 527)	5.7	2.0	5.7	0.1
naMCI_SD (*n* = 57)	5.6	2.0	5.7	0.3
naMCI_MD (*n* = 14)	4.4	2.3	4.5	0.5
aMCI_SD (*n* = 47)	5.8	1.9	6.0	0.3
aMCI_MD (*n* = 25)	4.3	1.6	4.2	0.4

The adjusted mean identification score included age, gender, year of schooling, mini-mental state examination (MMSE) score, hypertension, hyperlipidemia, diabetes, smoking history, alcohol consumption, geriatrics depression scales (GDS) score, and geriatrics anxiety inventory (GAI) score as covariates (ANCOVA, p < 0.05). NA, normal aging; naMCI_SD, non-amnestic MCI single domain; naMCI_MD, non-amnestic MCI multiple domain; aMCI_SD, amnestic MCI single domain; aMCI_MD, amnestic MCI multiple domain; n, no. of participants; SD, standard deviation; SE, standard error.

## Discussion

This study revealed the findings from a study of community-dwelling older adults in Singapore that measured smell identification performance using a locally developed nine-item smell test. The locally developed nine-item smell test was developed to deliver odor in a controlled manner by standardizing the concentration and amount of odor presented to participants upon sniffing. This standardization could allow us to quantitatively assess the smell ability of the study population. Our study indicated that the age-related smell identification impairment in older adults from age 60–80 years and above was correlated with age, in agreement with a previous meta-analysis study that found that the smell identification decline started at the age of 50 years (Zhang and Wang, 2017). Similar to previous studies, females outperformed males in the smell identification test (Mullol et al., 2012; Sorokowski et al., 2019). An earlier decrease in smell identification performance was more evident in males than females (Fornazieri et al., 2015).

Surprisingly, one of the findings of our study showed no significant difference between the NA and the overall MCI in smell identification score. This result contradicted a previous community-based cross-sectional study that reported MCI as a whole was associated with severely impaired smell identification compared with cognitively intact older adults. This previous study was limited to comparing NA and total MCI only and did no further investigation of the subtypes of MCI (amnestic and non-amnestic) and the number of affected memory or non-memory domain deficit (Liang et al., 2016). This rather contradictory result may be due to the heterogeneous memory or non-memory deficit of MCI diagnosis being combined as a whole MCI population to associate with smell identification impairment (Roalf et al., 2017).

Our study observed a significant difference between NA and aMCI_MD in the smell identification score, partially agreeing with a clinical study comparing smell identification among MCI subtypes. This clinical study showed a significant smell identification impairment between aMCI_MD and healthy control but not in other MCI subtypes (Lehrner et al., 2009). The inconsistency may be due to a smaller sample size collected for MCI subtypes in this previous study that could limit the statistical significance of the group comparisons. In both aMCI groups, we found a significant difference in smell identification impairment in aMCI_SD vs. aMCI_MD. This finding was aligned with similar results from previous clinical studies (Vyhnalek et al., 2015; Quarmley et al., 2017). A neuroimaging study may help to explain this finding. Brambati et al. (2009) found more widespread brain atrophy in gray matter in aMCI_MD than aMCI_SD. This report suggested that both aMCI single and multiple domains differed in degrees of severity transition from cognitively normal to AD. The smell identification impairment in aMCI_MD participants found in our study could support the association of smell impairment at baseline with a higher risk of developing probable AD or Lewy body dementia in their later life (Yoon et al., 2015). After controlling for relevant confounding variables, naMCI_SD significantly performed better than aMCI_MD. This is partially aligned with a previous study, which reported that both naMCI_SD and naMCI_MD performed better smell identification test than aMCI_SD and aMCI_MD groups (Park et al., 2018). Most of the studies reported the smell identification performance of naMCI group without further classification into single or multiple domain deficits (Lehrner and Deecke, 2000; Westervelt et al., 2008; Vyhnalek et al., 2015). Since this discrepancy has not been found elsewhere, it is probably due to varying diagnostic criteria for MCI, the types of neurocognitive tests used to assess the memory and non-memory deficits, and recruitment sources (clinical vs. community setting) (Roberts and Knopman, 2013).

Olfactory processing of smell identification task involved the olfactory cortex, orbital frontal cortex, and medial temporal lobes, typically neuropathological lesion sites for AD. The underlying mechanism of olfactory impairment in that early dementia could be due to greater brain activation to identify odor, which causes degenerative changes over time. These changes are associated with the atrophy of the entorhinal cortex and hippocampal volume (Murphy, 2019). A follow-up study showed that similar atrophy brain structures in MCI predicted conversion to AD (Devanand et al., 2007). A recent meta-analysis reported a predominant correlation between hippocampal atrophy and smell identification deficit in AD or MCI vs. healthy control (Su et al., 2021). Another supporting evidence from a review showed that hippocampal volume correlated with smell identification impairment in MCI from cross-sectional studies (Yi et al., 2022). Both reviews suggested that the olfactory test could be a supplementary tool for the early detection of cognitive decline with other predictors.

Our study results may help lay a foundation for a smell identification impairment in the aMCI multiple domain deficits group. It suggested that those diagnosed with aMCI multiple domain deficits were likely at more severe disease stages and had a higher risk of converting to AD later (Ding et al., 2016). A functional Magnetic Resonance Imaging (fMRI) study found that aMCI_SD participants had greater brain activation than healthy control in brain areas of language, memory, and attention, which could develop an early compensatory mechanism that reduced their cognitive deficit and was no different from cognitively intact older adults (Lenzi et al., 2011). This neuroimaging study could explain our finding that no smell identification difference exists between the aMCI_SD and NA and supports our finding that the aMCI_MD group had a significant degree of impairment in smell identification compared with aMCI_SD and NA older adults.

This study showed that older adults aged 80 years and above had a significant smell detection impairment. This result complements previous reports, demonstrating that smell detection was progressively impaired with aging (Mullol et al., 2012; Seow et al., 2016). Our study investigated smell detection and identification, which provide more details of smell ability than a single-staircase method (Seow et al., 2016). In addition, this study further confirmed that the smell detection impairment was not significantly different between the NA and all subtypes of MCI groups. However, with the current method of smell detection based on the presence of scent, these results might not be transferable to the smell detection threshold ability of older adults. Nonetheless, this finding is somewhat similar to a previous study that cognitive factors were not related to smell detection (Hedner et al., 2010). A meta-analysis manuscript reported that smell identification was more impaired than other smell test domains such as smell detection and smell memory in MCI participants (Roalf et al., 2017). A possible explanation for this might be that the smell detection ability depended on the peripheral olfactory processing and was not affected by cognitive impairment. A higher-order olfactory processing task such as smell identification and its impairment resulted from the neurodegenerative processes in the central nervous system (Hedner et al., 2010; Gros et al., 2017).

There are several limitations in this study, the first of which is that it was a cross-sectional study that did not allow for a causal relationship to be established. The study comprised a majority of women which could have a selection bias. The current smell tests integrating the neuropsychological test with a neuroimaging component in a future longitudinal study would allow us to predict the conversion of MCI to dementia and ascertain further whether the degree of smell identification impairment depends on the severity of the cognitive decline.

## Conclusion

Smell identification impairment was readily evident in participants suffering from aMCI_MD than in those with NA and single domain MCI. This suggests that olfactory impairment in the aMCI_MD deficits subgroup might be a potential baseline risk factor for predicting AD in later life. The locally developed smell test could benefit clinicians and researchers as a supplementary tool for identifying those with smell impairment at preclinical stages of cognitive decline, which could timely slow down or curb the progression to dementia.

## Data availability statement

The datasets presented in this article are not readily available because the datasets generated and/or analyzed for this study are not publicly available due to institutional regulations. Data is available from the first author (AY) on reasonable request but will be subjected to review and approval by NUS Institutional Review Board. Requests to access the datasets should be directed to AY, e0046890@u.nus.edu.

## Ethics statement

The studies involving human participants were reviewed and approved by the Institutional Review Board of the National University of Singapore. The patients/participants provided their written informed consent to participate in this study.

## Author contributions

AY collected and analyzed the data and drafted the manuscript. WZ and DW contributed to the data interpretation and guidance. RM, WZ, EK, and DW conceptualized the study, reviewed the results, advised on the manuscript preparation, and reviewed the manuscript. All authors read and approved the final version of this article.
